# Use of Guselkumab for the Treatment of Moderate-to-Severe Plaque Psoriasis: A 1 Year Real-Life Study

**DOI:** 10.3390/jcm9072170

**Published:** 2020-07-09

**Authors:** Marco Galluzzo, Lorenzo Tofani, Paolo Lombardo, Alessandra Petruzzellis, Dionisio Silvaggio, Colin Gerard Egan, Luca Bianchi, Marina Talamonti

**Affiliations:** 1 Dermatology Unit, Fondazione Policlinico, “Tor Vergata”, 00133 Rome, Italy; marco.galluzzo83@gmail.com (M.G.); lorenzotofani@hotmail.it (L.T.); lombardopaolo89@gmail.com (P.L.); alessandra.petruzzellis@gmail.com (A.P.); dionisio.silvaggio@gmail.com (D.S.); luca.bianchi@uniroma2.it (L.B.); 2 Department of Experimental Medicine, University of Rome “Tor Vergata”, 00133 Rome, Italy; 3 CE Medical Writing, 56023 Pisa, Italy; colingegan@gmail.com; 4 Department of Systems Medicine, University of Rome “Tor Vergata”, 00133 Rome, Italy

**Keywords:** Psoriasis, biological drugs, guselkumab, Psoriasis Area and Severity Index (PASI), real-life

## Abstract

Little information is available from real-life studies evaluating the efficacy of guselkumab in moderate-to-severe psoriasis. In this real-life study, we retrospectively examined a database of 52 patients with moderate-to-severe psoriasis treated with guselkumab (100 mg, s.c.) and followed for 1 year. Disease severity and treatment response was assessed by the Psoriasis Area and Severity Index (PASI) at baseline and after 4, 12, 20, 28, 36, 44, and 52 weeks. Predictors of a PASI response were evaluated by univariate and multivariate regression. After 12 months, 84.2% of patients (mean age 51.3 ± 14.1 years) treated with guselkumab achieved a PASI score of <3. Furthermore, PASI score decreased from 20 ± 13.3 at baseline to 4.4 ± 4.7 and 2.7 ± 3.9 at 12 and 20 weeks, and PASI 75, 90, and 100 response was achieved in 84.2%, 78.9%, and 63.2% of patients respectively at 12 months. Stepwise multivariate regression analysis revealed that previous biological treatment and the presence of comorbidities were associated with poorer response between 28–44 weeks, however the presence of obesity *per se* was not associated with poorer response. Difficult-to-treat areas were also improved as early as 12 weeks following guselkumab. Guselkumab was observed to be effective and safe in patients with moderate-severe chronic psoriasis in a real world-setting.

## 1. Introduction

Psoriasis is a chronic inflammatory immune skin disease affecting 2–3% of the world’s population [1]. Biological therapies such as tumor necrosis factor (TNF), interleukin (IL) 12/23, and IL-17 inhibitors have revolutionized the management of moderate-to-severe psoriasis, allowing a high proportion of patients to attain clear skin [2,3,4,5,6,7,8,9]. Despite this, many patients still remain untreated, do not respond, or experience treatment-related toxicities [10]. For patients with moderate-to-severe psoriasis, it is highly desirable to achieve adequate long-term disease control with continued administration of safe and effective treatments [11,12]. 

Guselkumab is an IL-23 inhibitor that binds to the p19 subunit of IL-23 that has been shown to be highly efficacious and well tolerated for the treatment of moderate-to-severe plaque psoriasis [7,8,13,14].

In two recent phase III studies, VOYAGE 1 [7] and VOYAGE 2, [8] guselkumab treatment (administered as a single 100 mg injection initially, 4 weeks later, and subsequently every 8 weeks) showed statistically significant improvements in efficacy compared with placebo, and superiority to adalimumab. In NAVIGATE, patients switched to guselkumab had superior improvement in psoriasis severity compared to patients maintained on ustekinumab up to 1 year [15]. In post-hoc analyses of the VOYAGE 1 and 2 trials, guselkumab was more effective than adalimumab, that was consistent across different body weight subgroups [16], similar to observations compared to secukinumab from the post-hoc analysis of the ECLIPSE trial [17]. Furthermore, difficult-to-treat areas, such as the palms and/or soles, also improved with guselkumab compared to adalimumab [18].

Differences exist between results derived from clinical trials and real-life daily practice using biological therapy for the treatment of psoriasis [19,20,21,22]. Furthermore, efficacy data evaluating the use of guselkumab in real-life clinical practice is limited, particularly over the long-term. In the present study, we have evaluated the efficacy of guselkumab in patients with moderate–severe chronic plaque psoriasis over 1 year in a real-world setting. 

## 2. Methods

### 2.1. Patients and Study Design

In this retrospective longitudinal study, we reviewed data from patients with moderate–severe chronic plaque psoriasis who were treated for at least 12 weeks (two doses) of guselkumab at our Dermatology Unit, “Tor Vergata" University of Rome from 1 November, 2018 to 31 March, 2020. Patients started treatment at different times during the study, so these data represent only a cross-sectional ‘snapshot’ of our experience taken up to the end of March 2020.

Guselkumab was administered in a standard dosing regimen (induction phase: 100 mg subcutaneously at weeks 0 and 4, and a maintenance dose every 8 weeks thereafter) to patients with moderate-to-severe psoriasis (Psoriasis Area Severity Index (PASI) >10) who failed to respond or had contraindications or side effects to at least one conventional treatment, including systemic therapy (i.e., methotrexate, cyclosporine, or acitretin) or phototherapy (i.e., ultraviolet B, psoralen plus ultraviolet A) according to Italian regulations for the prescription of biologic therapy [11]. Patients with a baseline PASI <10, who presented involvement of sensitive areas such as the face, scalp, hands, or genital areas were also considered eligible for treatment with guselkumab.

Exclusion criteria included patients with other autoimmune/inflammatory diseases such as Crohn’s disease, ulcerative colitis, rheumatoid arthritis, and ankylosing spondylitis; patients treated with a biologic <4 weeks or patients that had received systemic treatment or phototherapy in combination with biologic agent within 4 weeks of the first visit; and patients with guttate, erythrodermic, or pustular psoriasis. Diagnosis of psoriasis was clinical. Demographic and anamnestic data, comorbidities, and PASI score at the moment of enrolment were recorded using a dedicated database. 

All patients gave written informed consent for their participation prior to enrolment. This study complied with the ethical standards laid down in the 1975 Declaration of Helsinki.

### 2.2. Outcome Measures

Clinical efficacy was evaluated using PASI 75 90 100 (a 75%, 90%, 100% reduction in the PASI score) at the following time points: 4, 12, 20, 28, 36, 44, and 52 weeks. Primary inefficacy (failure to achieve PASI 75 after 16 weeks of treatment) or secondary inefficacy (loss of PASI 75 after 16 weeks of treatment) were also assessed.

### 2.3. Safety

Safety and tolerability (including the presence of any adverse events (AEs)) of guselkumab were evaluated over the duration of the study. Clinical laboratory tests and control of vital signs were also assessed.

### 2.4. Statistical Analysis

Data are presented as mean ± standard deviation for continuous variables, and number and percentage for categorical variables. Univariate regression for two parameters (bivariate analysis) was assessed by Pearson’s correlation coefficient. Stepwise multivariate logistic regression models were performed to evaluate the association between dependent variables (e.g., sex, age, age at onset of disease, disease duration, body weight, body mass index (BMI), PASI at baseline, number of comorbidities, and number of previous biologic treatments (PBT) on achievement of PASI 75, 90, and 100 at 4, 12, 20, 28, 36, 44, and 52 weeks and presented as odds ratio (OR) and 95% confidence intervals (CI).

Effectiveness data were analyzed using a last observation carried forward (LOCF) method, where if a patient dropped out of the study the last available value was ‘carried forward’ until the end of the treatment 23. *p* < 0.05 was considered statistically significant. All analysis was performed using STATA 11.2 software (Statacorp LP Inc., College Station, TX, USA.).

## 3. Results

### 3.1. Patient Demographic and Clinical Characteristics

In this real-life retrospective analysis, 52 patients with moderate-to-severe plaque psoriasis were treated with guselkumab and followed over a period of 12 months. Of the 52 patients who initiated treatment, 52 patients reached 12 weeks, 43 for 20 weeks, 40 for 28 weeks, 36 for 36 weeks, 31 for 44 weeks, and 15 for 52 weeks. Baseline characteristics of patients are presented in Table 1. The majority of patients were male (57.1%) with mean age of 51.3 ± 14.1 and long history of psoriasis (mean disease duration of 22.1 ± 16.2 years). Most patients were previously treated with a biological drug (*n* = 30, 57.7% and mean of 1.3 ± 1.5) and hypertension and obesity were the most frequent comorbidities observed (42.3% and 40.4%, respectively).

### 3.2. PASI Response

**Figure 1:**
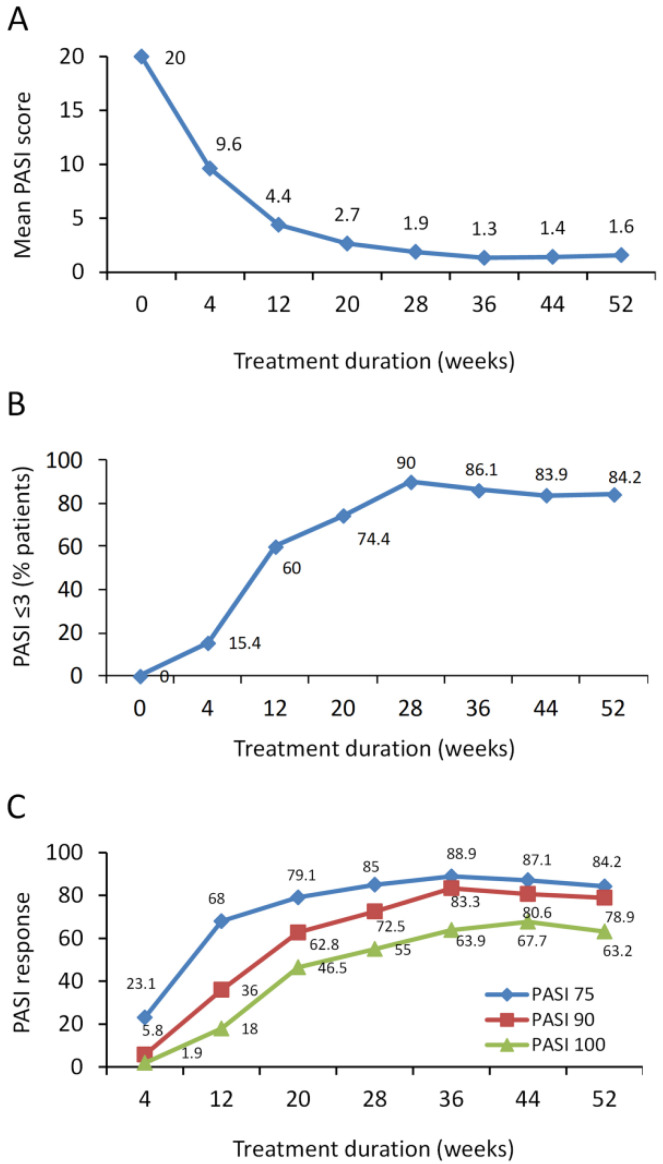
Effect of guselkumab in psoriatic patients on Psoriasis Area and Severity Index (PASI) score and achievement of PASI 75, 90, and 100 response over 1 year. (**A**) PASI is presented as mean values. (**B**) % patients achieving a PASI score ≤3 and (**C**) % patients achieving PASI 75, 90, and 100 response.

Guselkumab treatment decreased mean PASI score from 20 ± 13.3 at baseline to 4.4 ± 4.7 and 2.7 ± 3.9 at 12 and 20 weeks (Figure 1A). At 28 weeks, 90% of patients achieved a PASI score of ≤3 and this was maintained up to 1 year in 84.2% of patients (Figure 1B). At 12 weeks, PASI 75, 90, and 100 response was achieved in 68%, 36%, and 18% of patients, respectively, whereas at 20 weeks, PASI 75, 90, and 100 response was achieved in 79.1%, 62.8%, and 46.5% of patients, respectively (Figure 1C). At 1 year, PASI 75, 90, and 100 response was achieved in 84.2%, 78.9%, and 63.2% of patients, respectively. No patients were lost due to primary or secondary inefficacy (Figure 1C).

### 3.3. PASI Response in Different Patient Subgroups

Over the 1 year follow up, we identified specific best responder subgroups of patients. These included patients without comorbidities, biologic naïve patients, those with less than three PBTs, and patients who had less than three comorbid diseases and/or PBTs (combined).

**Figure 2:**
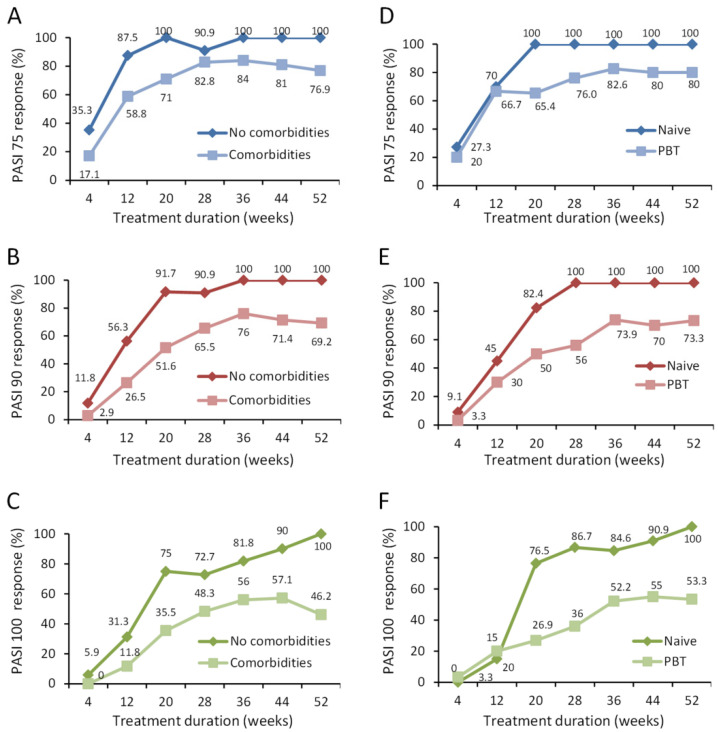
Effect of guselkumab in subgroups of psoriatic patients on achievement of PASI 75, 90, and 100 response over 1 year. (**A**–**C**) The % patients with comorbid diseases (*n* = 35) compared to those without comorbid diseases (*n* = 17) in achieving PASI 75 (**A**), 90 (**B**), and 100 (**C**) response are presented for each time point. (**D**–**F**) The % patients naïve to previous biological treatment (*n* = 22) compared to those having previously received biological treatment (PBT; *n* = 30) in achieving PASI 75 (**D**), 90 (**E**), and 100 (**F**) response are presented for each time point. The % of patients at each time point is shown.

**Figure 3:**
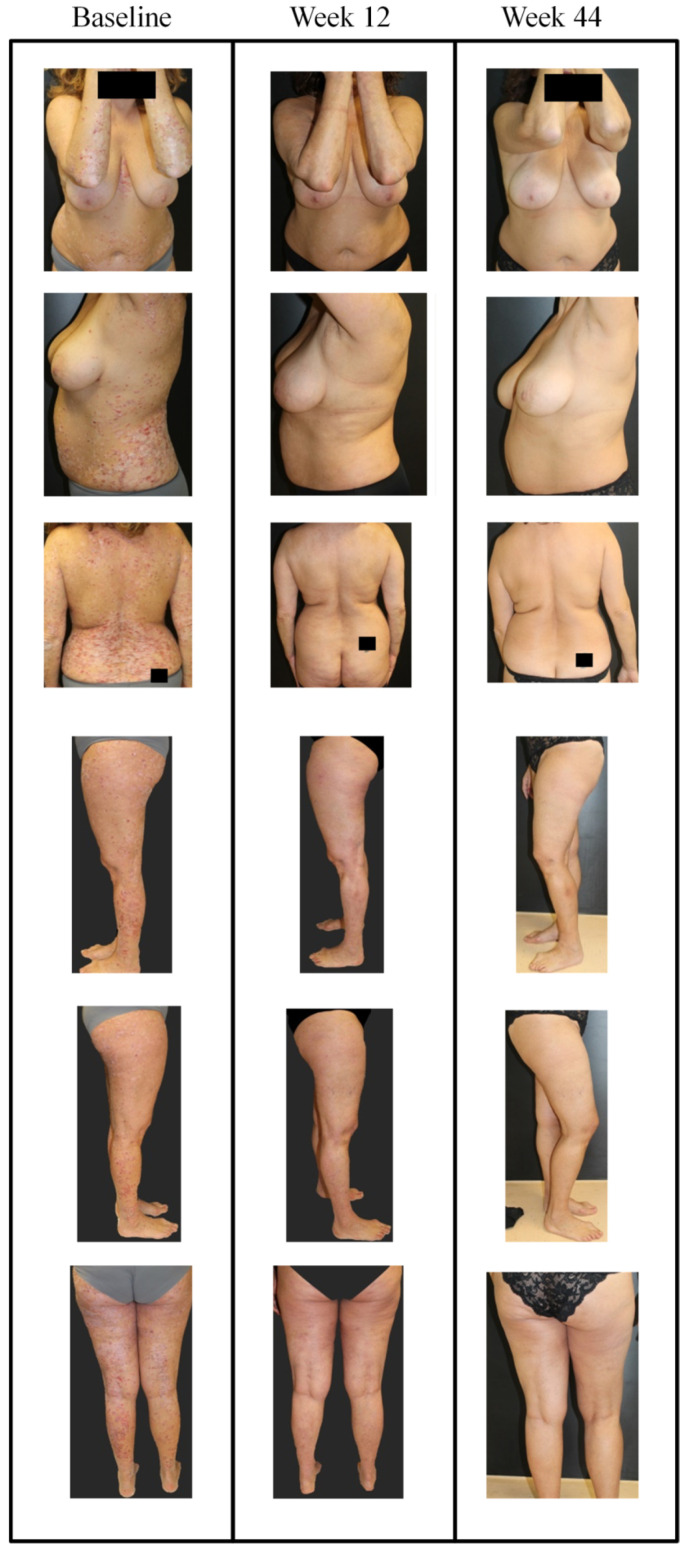
Representative images of outcome in case #1; a female patient, aged 52 years with onset of psoriasis at 47 years of age, cigarette smoker (34 pack years) with dyslipidemia and naïve to biological treatment. In January 2019 she presented with severe psoriasis and arthralgia in the small joints of the hands. Ultrasound examination in this patient identified dactylitis and moderate tenosynovitis associated with weak but positive color Power Doppler signal which corresponded with the proximal and distal phalangeal joints of the second finger of the right hand. Previous therapies included cyclosporine (250 mg/day) from 2014 to December 2018, without interruption, with loss of response in the last 3 months. Biological screening tests did not reveal any noteworthy alterations. Images at different sites are presented for the time points; baseline, week 16, and week 36. After baseline visit (PASI of 44.4 and visual analogue scale (VAS) pain of 80), guselkumab treatment was initiated (induction phase: 100 mg subcutaneously at weeks 0 and 4, and a maintenance dose every 8 weeks thereafter). After 12 weeks, PASI decreased to 3 and VAS pain was 30; at week 44, PASI decreased further to 0 and pain VAS was 20.

**Figure 4:**
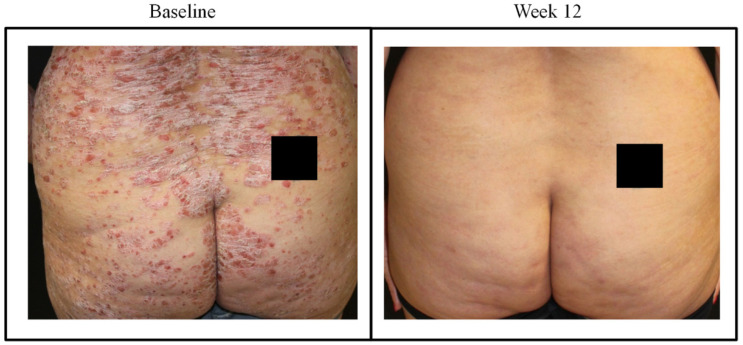
Representative images of outcome at the gluteal region in case #1; a female patient, aged 52 years with onset of psoriasis at 47 years of age. At baseline, PASI was 44.4 and VAS pain was 80. After 12 weeks with guselkumab (induction phase: 100 mg subcutaneously at weeks 0 and 4, and a maintenance dose every 8 weeks thereafter), PASI decreased to 3 and VAS pain was 30.

Patients without comorbidities showed 100% PASI response at 1 year, whereas PASI 75, 90, and 100 response for patients with comorbid diseases was 76.9%, 69.2%, and 46.2% respectively (Figure 2A–C). In biologic naïve patients, 100% response was achieved for PASI 75, 90, 100 vs. 80%, 73.3%, and 53.3% respectively in those with PBT at 1 year (Figure 2D–F). This improvement is also depicted in images of a female patient naïve to biological treatment where as early as 12 weeks, guselkumab treatment decreased PASI score from 44 to 3 (Figure 3 and Figure 4).

**Figure 5:**
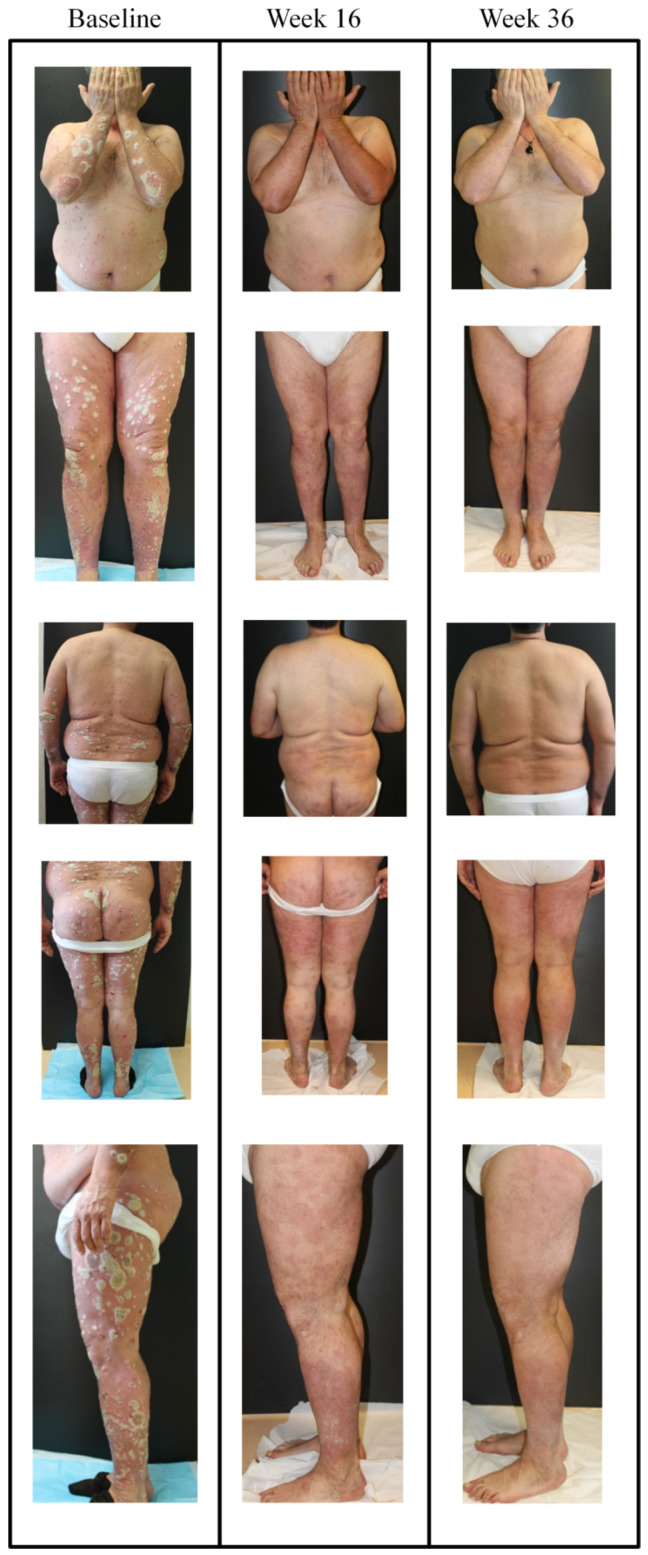
Representative images of outcome in case #2; male, aged 53 years with onset of psoriasis at 24 years of age, cigarette smoker with hypertension. In January 2019 he presented with severe chronic plaque psoriasis. He was not responsive to traditional systemic drugs and anti-TNFα (i.e., multidrug resistant). He was treated with secukinumab from 2016 to 2019, with excellent response over the first year of treatment but loss of efficacy occurred from week 64 of treatment, at which point he was switched to guselkumab. Images at different sites are presented for the time points; baseline, week 16, and week 36. After baseline visit (PASI of 42, guselkumab treatment was initiated (induction phase: 100 mg subcutaneously at weeks 0 and 4, and a maintenance dose every 8 weeks thereafter). After 16 weeks, PASI decreased to 3 and at week 36, PASI decreased to 0.

In patients with <3 PBTs, PASI 75, 90, and 100 response was 87.5%, 87.5%, and 75%, respectively, vs. 71.4%, 57.1%, and 28.6%, respectively, in patients treated with ≥3 PBTs (Figure S1). Images showing improvement following guselkumab treatment in a patient with hypertension who was not responsive to traditional systemic drugs, anti-TNFα, or anti-IL17A treatment is shown in Figure 5.

Stratifying patients with low PBTs and few comorbid diseases (0–1 for either), the improvement in PASI response was amplified: 100% of patients achieved PASI 75, 90, and 100 response compared to patients having ≥3 PBTs and/or comorbid diseases where the PASI response was substantially reduced (72.7%, 63.6%, and 36.4% for PASI 75, 90, and 100, respectively; Figure S2). Combining the number of PBTs and/or comorbid disease together in patients we observed a positive correlation, whereby as the number of PBTs or comorbid diseases increased, so too did PASI score at 12 weeks (Figure S3A). As expected, the inverse was observed when the number of patients with PBTs and/or comorbid diseases was correlated with an improvement in PASI score at 2 weeks (Figure S3B).

### 3.4. Predictors of Improved PASI Response

Univariate logistic regression was next used to identify potential predictors of PASI response. Variables included in the model were sex, age, age at onset of disease, disease duration, body weight, BMI, PASI at baseline, number of comorbidities, number of PBTs, and number of comorbidities and PBTs. For PASI 75 response, low number of PBTs, comorbid diseases, and number of comorbidities and PBTs combined, were associated with improved PASI 75 response at 20 weeks (Table 2).

These same three variables were also associated with an improved PASI 90 response at 12, 20, 28, 36, and 44 weeks, with absence of or low number of comorbidities emerging as the strongest predictor odds ratio (OR of 4.76 at 44 weeks; Table 3). Similar to PASI 90 response, these three variables also emerged as predictors of complete remission (PASI 100 response), at 20, 28, 36, 44, and 52 weeks (Table 4).

Stepwise multivariate logistic regression was next applied to determine predictors of PASI response using the same covariates in the model. Similar to univariate analysis, both the number of PBTs (low) and comorbidities (low) emerged as being predictors of improved PASI response. In particular, a low number of comorbidities was strongly associated with PASI 90 response at 36 and 44 weeks as well as PASI 100 response at 44 and 52 weeks (Table 5). We also initially included the variable: number of comorbid diseases and number of PBTs in the model, but it was subsequently excluded due to collinearity.

### 3.5. Safety

Evaluation of the safety and tolerability for the duration of the study revealed that treatment with guselkumab was generally well tolerated without evidence of cumulative toxicity or organ toxicity. No patients dropped out of the study due to AEs. Clinical laboratory tests and control of vital signs showed no significant alteration.

### 3.6. Difficult-to-Treat Locations

**Figure 6:**
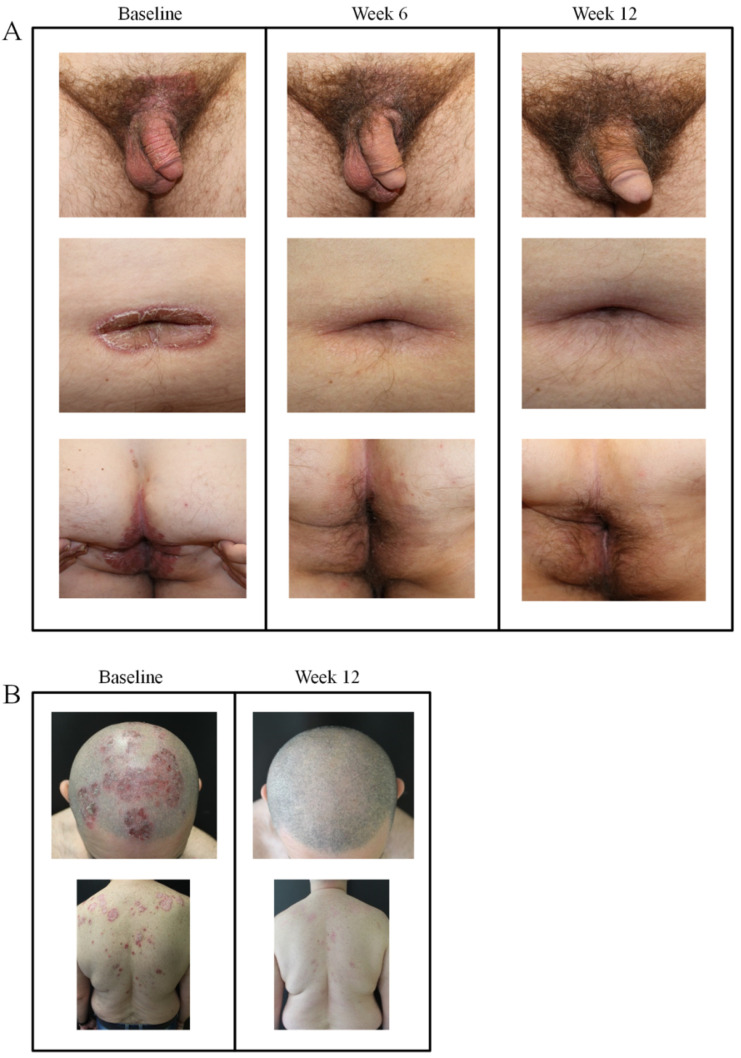
Plaque psoriasis in difficult-to-treat locations before and after initiation of guselkumab therapy. (**A**) Patient with psoriasis at genital (suprapubic area, penis foreskin perianal) and umbilicus/periumbilical areas at baseline and after 6 and 12 weeks of treatment. (**B**) Patient with head, scalp, and shoulder plaques prior to initiating guselkumab and after 12 weeks of therapy.

Representative images of two cases with moderate-to-severe plaque psoriasis in difficult-to-treat locations treated with guselkumab are shown in Figure 5. In a male patient with psoriasis in the suprapubic area and penis foreskin, umbilicus/periumbilical, and perianal regions, treatment with guselkumab led to complete clearance of plaques by 12 weeks (Figure 6A). A male patient with plaque psoriasis of the scalp and upper shoulder region also showed almost complete clearance after 12 weeks with guselkumab (Figure 6B).

## 4. Discussion

Guselkumab is the first anti-IL-23 p19 antibody approved by the Food and Drug Administration and European Medical Agency for the management of moderate-to-severe psoriasis in adults [23,24]. However, limited post-marketing data are currently available on its effectiveness and tolerance. IL-23 inhibitors are effective when dosed every 8–12 weeks compared to every 2 weeks for adalimumab, or 4 weeks for IL-17 blockers. With only six administrations after the first year, guselkumab is more “comfortable” compared to TNF-α and IL-17 [23,24]. 

In this retrospective study, we analyzed data from a total of 52 patients with moderate-to-severe psoriasis treated with guselkumab in daily clinical practice up to 1 year who either had not responded, or had contraindications or were intolerant to traditional systemic therapies or who had not responded to anti-TNFα, anti-IL-17, or anti-IL-12/IL-23 biological drugs.

PASI 90 response values obtained from our real-life analysis of patients with moderate-to-severe psoriasis corroborate with results observed for VOYAGE and ECLIPSE trials, where we observed 72.5% at 28 weeks and 80.6% at week 44 compared to 80.6% at week 24 and 76.3% at week 48 in VOYAGE 1 and 84.5% at week 48 in ECLIPSE. Achievement of PASI 75 response was also in line with those from these trials, particularly at time points close to 1 year (87.8% at 48 weeks in VOYAGE 1 compared to 87.1% at week 44 in our study). The proportion of patients achieving complete remission at early as well as later time points was superior in the present study compared to these trials. In VOYAGE 1 at week 24, PASI 100 response was achieved in 44.4% of patients, remaining relatively unchanged up to 48 weeks (47.4%). In the present study, 46.5% of patients achieved complete remission at 20 weeks, increasing to 55% at 28 weeks, and 63.9% at 36 weeks remaining stable up to 52 weeks. A slightly higher proportion of patients achieved PASI 100 response at 48 weeks in ECLIPSE (58.2%) [25]. One important limitation of these trials was the fact that patients had to comply with strict inclusion and exclusion criteria that are not commonly found in patients in real-life practice [26].

The efficacy of guselkumab treatment was also recently evaluated in two real-life studies [27,28]. In the first study performed in Spain, 55 patients with moderate-to-severe plaque-type psoriasis were treated with guselkumab over a period of 36 weeks [27]. While 100% of patients achieved a PASI 75 response at 36 weeks (compared to 87.1% in our study), about 75% achieved PASI 90 response compared to 80.6% in our study and about 55% achieved complete remission (67.7% in the present study). Variables such as gender, PBTs, disease duration, and comorbidities were similar to those in our study. However, the greater efficacy observed at later time points for PASI 90 and PASI 100 response may be attributed to higher baseline PASI in our study (20 vs. 13.7) as previously shown in the real-life BioCAPTURE cohort, where a baseline PASI ≥10 was found to be a strong predictor for achieving PASI 90 [29]. In the second study, undertaken in France, the effectiveness and tolerance of guselkumab for psoriasis under real-life conditions was also evaluated in a multi-center study up to 16 weeks [28]. Overall, 38.3% of patients achieved PASI 100 at week 16 and 50.6% achieved PASI 90. 

While we did not evaluate PASI response at 16 weeks in our study, we can estimate that between 12 and 20 weeks (16 weeks), the PASI 90 and 100 response was achieved in 49.3% and 32.3% of patients respectively, a level of improvement that was closely comparable. It is important to highlight that patients in our real-life study had a baseline PASI of 20 ± 13.3 vs. 12.7 ± 8.95 and 13.7 ± 7.7 in the French and Spanish study, respectively, and the level of improvement attained was consistent at these early time points and maintained up to 1 year. Together, real-life experience using guselkumab in Spain [27], France [28], and findings reported here in Italy, confirm the early, rapid, and sustained effectiveness of guselkumab. Findings from these real-life studies confirm data derived from VOYAGE 1 and 2 [7,8], despite differences in patient characteristics such as disease severity at baseline, exposure to PBTs, and gender, already noted [28]. 

While the efficacy results observed in the present study were indeed favorable, subgroup analysis allowed us to identify best responder patients who could benefit to an even greater extent. These populations were namely patients without the burden of comorbid diseases or naïve to PBTs or who had few PBTs. In both of these patient populations, we observed complete remission at 52 weeks compared to approximately 50% of patients achieving complete remission with comorbidities or those with PBTs. Indeed, these two variables strongly emerged in both univariate and multivariate analysis. 

The presence of obesity did not emerge as a predictor of PASI response in either univariate or multivariate analysis in this small cohort. Confirming this, and despite obese patients having a higher baseline PASI than non-obese patients (24.6 ± 14.3 vs. 17.9 ± 12.6 respectively), at 12 weeks, PASI levels in both groups of patients were decreased to a similar extent (5.9 ± 5.2 vs. 3.6 ± 4.2) and further still at 20 weeks (3 ± 4.9 vs. 2 ± 3.1), stabilizing thereafter. Indeed, in post-hoc analyses of the VOYAGE 1 and 2 trials, guselkumab showed improved efficacy over adalimumab, that was consistent across different body weight subgroups [30], similar to observations compared to secukinumab from the post-hoc analysis of the ECLIPSE trial [17]. This favorable efficacy profile observed by guselkumab in obese patients is not shared across other classes of biological agents. We have previously observed that the absence of obesity (BMI ≤30) emerged as a good predictor of treatment response in our real-life analysis using ustekinumab [9]. 

Confirming these observations, Naldi and colleagues analyzed data from dermatology clinics in psoriasis patients receiving different biologics and observed that PASI 75 response decreased with increasing BMI [31]. A recent meta-analysis by Mourad et al. comprising 16 cohort studies and a total of 32,194 patients, demonstrated that obesity predicted lower rates of biologic persistence due to ineffectiveness in etanercept, ustekinumab, and infliximab groups [32]. Post-hoc analyses of VOYAGE 1 and 2 trials also demonstrated that guselkumab improved efficacy over adalimumab across different body weight subgroups, particularly those weighing ≥100 kg [30]. Lack of effectiveness in obese patients may be attributed to lower blood drug levels as well as higher body surface area or increased levels of adipose tissue-derived pro-inflammatory cytokines [33,34]. Considering that over one-third of patients with moderate-to-severe psoriasis can also be obese [16,35,36], and also in real-life clinical practice [27,28], the availability of biologics such as guselkumab undoubtedly offer significant benefit in these severe patients over several other biologics.

Furthermore, a secondary analysis of the VOYAGE 1 and 2 trials showed that guselkumab improved difficult to treat areas such as the palms and/or soles compared to adalimumab [18]. Similar observations were also made in the present study where difficult to treat locations such as the genital region and scalp and shoulder region led to almost complete skin clearance by 12 weeks.

A limitation of the present study was the low number of patients (*N* = 52) and the relatively brief follow-up period (1 year). However, this biologic has only recently become available, limiting the possibility of attaining longer treatment periods. Since the majority of patients who participated in this study failed to respond to traditional systemics or other biologics, it would have been difficult to have a comparator group. Patients with other autoimmune/inflammatory diseases, necessitating other treatments were excluded to avoid confounding, thereby potentially detracting from the applicability of this cohort to the general population. We did not ask patients to complete a questionnaire to determine their level of satisfaction and/or quality of life. We certainly feel that this aspect needs to be formally explored in a future study with larger sample size and longer follow-up period.

## 5. Conclusions

In the present study, guselkumab was shown to be extremely effective for the treatment of moderate-to-severe real-life chronic plaque psoriasis in a real-life clinical practice over 1 year. We observed the best response of this biologic in patients without comorbid diseases and those naïve to or treated with few PBTs. Patients suffering from psoriasis in difficult to treat areas also benefited from guselkumab. Additional long-term studies with greater sample size are warranted to verify these preliminary findings.

## Figures and Tables

**Table 1:** Baseline clinical characteristics of psoriasis patients.

Clinical Characteristic	*N* = 52
General	
Male gender, *n* (%)	30 (57.7)
Age (years)	51.3 ± 14.1
BMI (Kg/M^2^)	29.9 ± 6.3
Current cigarette smoker, *n* (%)	25 (48)
Disease characteristics	
Age at disease onset	29.1 ± 15.9
Disease duration	22.1 ± 16.2
PASI at baseline	20.0 ± 13.3
Biologic therapy, *n* (%)	
Biologic naïve	22 (42.3)
1 biologic	9 (17.3)
2 biologics	10 (19.2)
≥3 biologics	11 (21.1)
Comorbidities, *n* (%)	
Hypertension	22 (42.3)
Obesity	21 (40.4)
Dyslipidemia	8 (15.4)
Psychiatric illness	3 (5.8)
Diabetes mellitus	3 (5.8)
Other pathologies/disorders	6 (11.5)

BMI, body mass index; PASI, Psoriasis Area and Severity Index. Data presented as mean ± standard deviation or number and %.

**Table 2:** Univariate logistic regression analysis of variables associated with PASI 75 response.

Variable	OR (95% CI)	*p*-Value
20 weeks		
Number of PBT	2.86 (1.41–5.88)	0.004
Number of comorbidities	2.56 (1.14–5.88)	0.023
PBT + comorbidities	1.96 (1.27–3.03)	0.003

CI, confidence interval; OR, odds ratio; PASI, Psoriasis Area Severity Index; PBT, previous biologic treatment. Variables included in model were sex, age, age at onset of disease, disease duration, body weight, BMI, PASI at baseline, number of comorbidities and number of previous biologic treatments.

**Table 3:** Univariate logistic regression analysis of variables associated with PASI 90 response.

Variable	OR (95% CI)	*p*-Value
12 weeks		
Number of PBT	1.96 (1.14–3.45)	0.017
Number of comorbidities	2.00 (1.03–3.85)	0.041
PBT + comorbidities	1.69 (1.11–2.56)	0.015
20 weeks		
Number of PBT	2.00 (1.19–3.33)	0.008
Number of comorbidities	2.94 (1.32–6.25)	0.008
PBT + comorbidities	1.85 (1.22–2.78)	0.004
28 weeks		
Number of PBT	2.17 (1.22–3.85)	0.008
Number of comorbidities	2.44 (1.14–5.26)	0.023
PBT + comorbidities	1.75 (1.18–2.56)	0.005
36 weeks		
Number of PBT	2.17 (1.12–4.17)	0.021
Number of comorbidities	5.26 (1.37–20.00)	0.015
PBT + comorbidities	1.96 (1.20–3.23)	0.007
44 weeks		
Number of PBT	2.04 (1.18–4.17)	0.014
Number of comorbidities	4.76 (1.33–16.67)	0.016
PBT + comorbidities	1.89 (1.16–3.03)	0.010

CI, confidence interval; OR, odds ratio; PASI, Psoriasis Area Severity Index; PBT, previous biologic treatment. Variables included in model were sex, age, age at onset of disease, disease duration, body weight, BMI, PASI at baseline, number of comorbidities, and number of previous biologic treatments.

**Table 4:** Univariate logistic regression analysis of variables associated with PASI 100 response.

Variable	OR (95% CI)	*p*-Value
20 weeks		
Number of PBT	2.86 (1.47–5.88)	0.002
Number of comorbidities	2.78 (1.30–5.88)	0.008
PBT + comorbidities	2.63 (1.39–5.00)	0.003
28 weeks		
Number of PBT	2.22 (1.27–4.00)	0.006
Number of comorbidities	2.38 (1.15–5.00)	0.019
PBT + comorbidities	1.89 (1.20–2.94)	0.006
36 weeks		
Number of PBT	2.04 (1.16–3.70)	0.013
Number of comorbidities	2.78 (1.23–6.25)	0.014
PBT + comorbidities	1.82 (1.18–2.78)	0.007
44 weeks		
Number of PBT	2.22 (1.18–4.17)	0.014
Number of comorbidities	3.33 (1.30–9.09)	0.012
PBT + comorbidities	1.89 (1.19–2.94)	0.007
52 weeks		
Number of PBT	2.63 (1.08–6.25)	0.034
Number of comorbidities	6.67 (1.25–33.33)	0.026
PBT + comorbidities	2.13 (1.10–4.17)	0.025

CI, confidence interval; OR, odds ratio; PASI, Psoriasis Area Severity Index; PBT, previous biologic treatment. Variables included in model were sex, age, age at onset of disease, disease duration, body weight, BMI, PASI at baseline, number of comorbidities and number of previous biologic treatments.

**Table 5:** Multivariate logistic regression analysis (stepwise analysis) of predictors of PASI 75, 90, and 100 response after treatment with guselkumab.

Variable	OR (95% CI)	*p*-Value
PASI 75		
20 weeks		
Number of PBT (low to high)	2.85 (1.41–5.88)	0.004
PASI 90		
12 weeks		
Number of PBT (low to high)	1.96 (1.14–3.45)	0.017
20 weeks		
Number of comorbidities (low to high)	2.94 (1.32–6.25)	0.008
28 weeks		
Number of PBT (low to high)	2.17 (1.22–3.85)	0.008
36 weeks		
Number of comorbidities (low to high)	5.26 (1.37–20.00)	0.015
44 weeks		
Number of comorbidities (low to high)	4.76 (1.33–16.67)	0.016
PASI 100		
20 weeks		
Number of PBT (low to high)	2.86 (1.47–5.88)	0.002
28 weeks		
Number of PBT (low to high)	2.22 (1.27–4.00)	0.006
36 weeks		
Number of PBT (low to high)	2.04 (1.16–3.70)	0.013
44 weeks		
Number of comorbidities (low to high)	3.33 (1.30–9.09)	0.012
52 weeks		
Number of comorbidities (low to high)	6.67 (1.25–33.33)	0.026

CI, confidence interval; OR, odds ratio; PASI, Psoriasis Area Severity Index; PBT, previous biologic treatment.

## Supplementary Materials

The following are available online at https://www.mdpi.com/2077-0383/9/7/2170/s1, Figure S1: Effect of guselkumab in psoriatic patients on achievement of Psoriasis Area Severity Index (PASI) 75, 90, and 100 response in patient subgroups over 1 year: effect of <3 (*n* = 19) or ≥3 previous biologic treatment (PBTs; *n* = 11), Figure S2: Effect of guselkumab in psoriatic patients on achievement of PASI 75, 90, and 100 response in patient subgroups over 1 year: effect of combined PBTs and comorbid diseases, Figure S3: Association between the number of PBTs and/or comorbid diseases on PASI score or improvement in PASI score at 12 weeks.
