# Supplementary material for: Use of Guselkumab for the Treatment of Moderate-to-Severe Plaque Psoriasis: A 1 Year Real-Life Study
Source: J Clin Med. 2020 Jul 9;9(7):2170. doi: 10.3390/jcm9072170 (PMC7408922; doi:10.3390/jcm9072170)
Supplement: Supplementary file 1 [file jcm-09-02170-s001.pdf]

Supplementary Materials: Use of Guselkumab for the Treatment of Moderate-to-Severe Plaque Psoriasis: a 1 Year Real-Life Study

Marco Galluzzo, Lorenzo Tofani, Paolo Lombardo, Alessandra Petruzzellis, Dionisio Silvaggio, Colin Gerard Egan, Luca Bianchi and Marina Talamonti

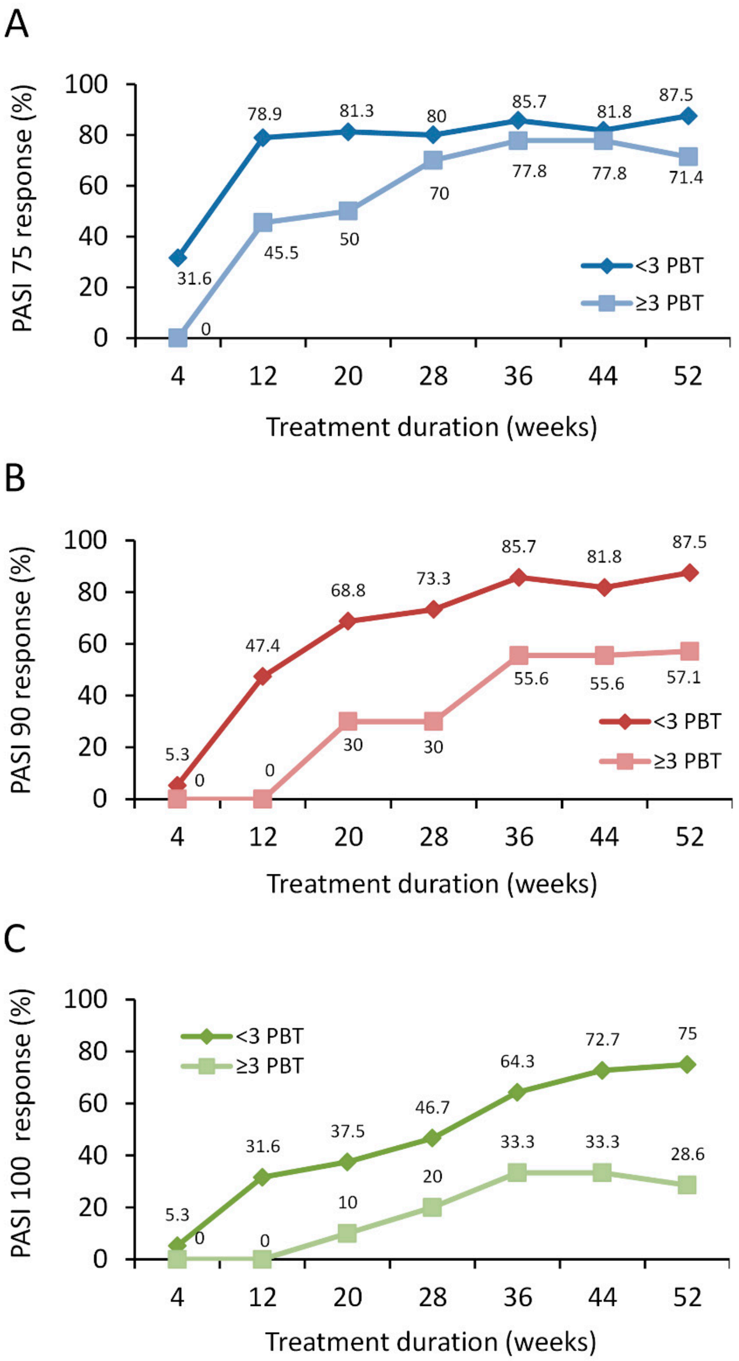

Figure S1. Effect of guselkumab in psoriatic patients on achievement of PASI 75 (**A**), 90 (**B**) and 100 (**C**) response in patient subgroups over 1 year: effect of <3 (N = 19) or ≥3 PBTs (N = 11). The % patients achieving PASI 75, 90 and 100 response are presented for each time point. The % of patients at each time point is shown.

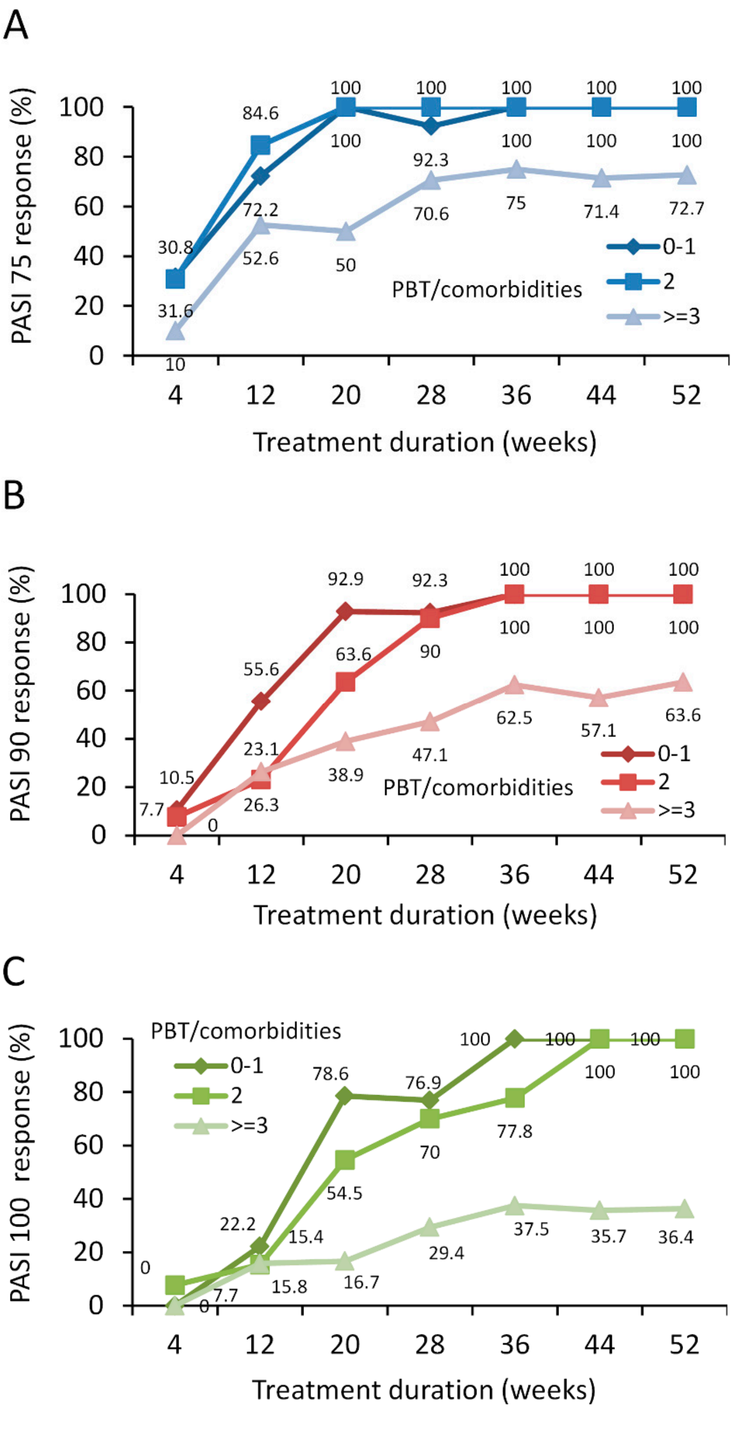

Figure S2. Effect of guselkumab in psoriatic patients on achievement of PASI 75 (**A**), 90 (**B**) and 100 (**C**) response in patient subgroups over 1 year: effect of combined PBTs and comorbid diseases. The % patients achieving PASI 75, 90 and 100 response are presented for each time point. 0–1 PBT/comorbidities (*N* = 19); 2 PBT/comorbidities (*N* = 13); ≥3 PBT/comorbidities (*N* = 20). The % of patients at each time point is shown.

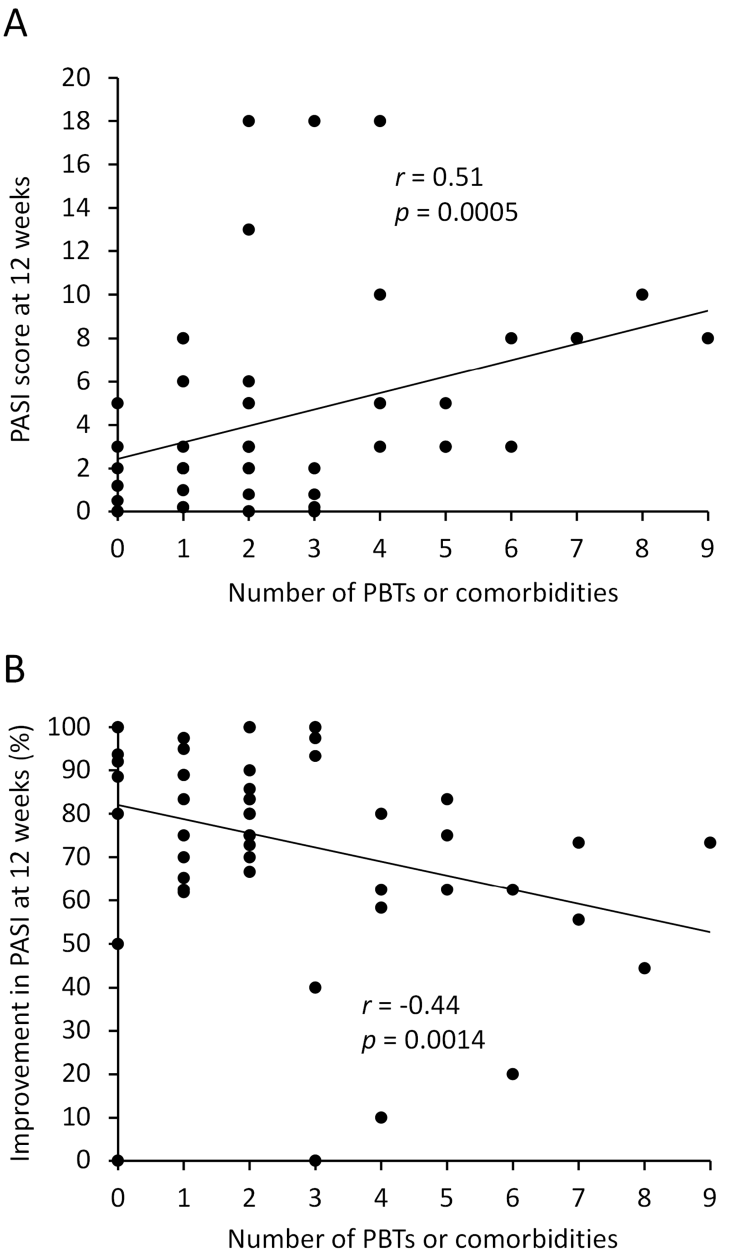

Figure S3. Association between the number of PBTs and/or comorbid diseases on PASI score or improvement in PASI score at 12 weeks. (**A**) The number of patients with PBTs and/or comorbid diseases positively correlates ($r = 0.51$, $p = 0.0005$) with PASI score at 12 weeks; (**B**) the inverse being observed ($r = -0.44$, $p = 0.0014$) when the number of patients with PBTs and/or comorbid diseases is plotted against improvement in PASI (%) at 12 weeks (compared to baseline).
